# Adjuvant Treatment of Crohn's Disease with Traditional Chinese Medicine: A Meta-Analysis

**DOI:** 10.1155/2019/6710451

**Published:** 2019-03-05

**Authors:** Yue Wang, Ming Li, An-Sheng Zha

**Affiliations:** ^1^Department of Gastroenterology, The First Affiliated Hospital of Anhui University of Traditional Chinese Medicine, Hefei 230031, China; ^2^Nanjing University of Traditional Chinese Medicine, Nanjing 210000, China

## Abstract

The objective of the meta-analysis was to evaluate the efficacy and safety of Traditional Chinese Medicine (TCM) in the treatment of Crohn's disease (CD). Pubmed, Embase, Medline, Web of Science, China National Knowledge Infrastructure, Chinese Biomedical Literature, Wanfang Database, and Cochrane Central Register of Controlled Trials were searched (through October 2018). The quality of randomized clinical trials meeting the inclusion criteria was assessed and the data were extracted according to the Cochrane Review Handbook v5.0 by two evaluators. A meta-analysis was performed using the software Stata 12.0. Twelve randomized controlled trials (RCTs) were selected. The studies were of low methodological quality. The meta-analysis indicated that treatment with TCM and Western Medicine (WM) was significantly superior compared to treatment with WM alone with regard to total effective rate, remission maintenance rate, reduction of C-reactive protein (CRP), reduction of erythrocyte sedimentation rate (ESR), clinical score reduction, and reduction of adverse events. Mucosal healing was improved in both the TCM-WM and WM groups; however, there were no significant differences between the two groups. There was a certain publication bias in the studies with regard to efficiency, adverse reactions, mucosal healing, and recurrence rate; however, there was no obvious publication bias with regard to other indicators. TCM, as an adjuvant therapy with WM, shows advantages in inducing remission in CD. The current evidence suggests that TCM-WM treatment might be more efficient in terms of total effective rate, remission maintenance rate, CRP reduction, ESR reduction, clinical score reduction, and reduction of adverse events than treatment with WM alone. Because of the low quality of the included RCTs, high quality confirmatory evidence is needed to assess the clinical value of TCM in the treatment of CD.

## 1. Introduction

Crohn's disease (CD) is a chronic inflammatory condition of the gastrointestinal tract, characterized by symptoms of abdominal pain, diarrhea, and fatigue [1]. It can affect any portion of the digestive tract including the mouth, esophagus, and small and large intestines, but is most common in the ileum. The incidence of CD has markedly increased in recent years. The pathophysiological mechanisms responsible for the development of CD remain unclear. It is widely accepted that the pathogenesis of CD likely involves genetic, environmental, and immunological factors [2, 3].

Currently, there is no cure for CD. Treatment is focused on relieving the symptoms and inducing remission [1]. Drugs such as 5-aminosalicylic acid, glucocorticoids, and immunosuppressive agents provide symptomatic relief. However, the use of these drugs is often associated with poor efficacy, treatment-related side effects, and long-term toxicity [4].

Recent studies have shown that Traditional Chinese Medicine (TCM) may have some advantages in the treatment of CD [5]. Many clinical studies have been reported with regard to the therapeutic effects of TCM on CD, but they lack the support of evidence-based medicine. Therefore, we have conducted a systematic review to evaluate the efficacy and safety of TCM in treating CD and to provide a scientific basis for its clinical use.

## 2. Methods

### 2.1. Databases Searched

We searched Pubmed, Embase, Medline, Web of Science, Cochrane China National Knowledge Infrastructure, Chinese Biomedical Literature, and the Wanfang Database for studies prior to October 2018. The search terms used were “CD” or “IBD” and “TCM”. There were no language restrictions.

### 2.2. Inclusion Criteria

The inclusion criteria were as follows: (1) randomized controlled trials (RCTs) or quasi-RCTs; (2) studies where patients met the diagnostic criteria of CD; (3) studies that assessed therapeutic effects including one or more parameters, such as total effective rate, mucosal healing, clinical score, ESR, CRP, remission maintenance rate, and side effects; and (4) studies where patients were treated with Western Medicine (WM) in the control group, and with TCM alone or TCM in combination with WM in the experimental group.

### 2.3. Exclusion Criteria

The exclusion criteria were as follows: (1) animal experiments, (2) duplicate studies, (3) studies with incomplete data, and (4) studies where the baseline data were not similar.

### 2.4. Data Extraction and Quality Evaluation

The data were extracted independently by two reviewers for baseline information, total effective rate, mucosal healing, clinical score, erythrocyte sedimentation rate (ESR), C-reactive protein (CRP), remission maintenance rate, and side effects. The two investigators independently assessed the methodological quality by using the quality assessment criteria described in version 5.0.2 of the Cochrane Handbook for Systematic Reviews of Interventions [6]. Where disagreements occurred, the opinion of a third investigator was obtained. The studies were assigned grades of A, B, and C based on the following criteria: randomization, allocation concealment, blinding, incomplete outcome data, selective reporting, and other biases. Grade A studies fully conformed to the six quality standards and had the lowest possibility of bias. Grade B partially conformed to one or more quality standards and had a moderate possibility of bias. Grade C did not conform to any of the six quality standards and had a high possibility of bias.

### 2.5. Data Analysis

Meta-analysis was performed using the software Stata (Version 12.0; STATA Corporation, College Station, TX, USA). Heterogeneity with regard to dichotomous data (total effective rate, mucosal healing, recurrence rate, and side effects) was evaluated using relative risk (RR), and with regard to continuous data (clinical score, ESR, and CRP) using the standardized mean difference (SMD). All RRs and SMDs were indicated with 95% confidence intervals (95%* CI*). We used the *χ*^2^ statistic and* I*^2^ test to analyze the heterogeneity across studies. Data were synthesized using a fixed-effect model; however, for a value of* I*^2^ > 50%, which was considered to indicate heterogeneity, a random-effect model was used. Egger's and Begg's regression tests were used to assess publication bias.

## 3. Results

### 3.1. Characteristics of the Studies

**Figure 1:**
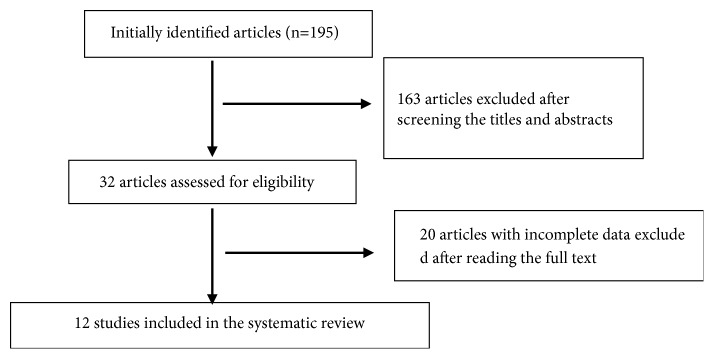
Flowchart showing results of literature search.

The initial search yielded 195 articles. After reading titles and abstracts, 183 articles that did not conform to the inclusion criteria were excluded. Finally, 12 articles [7–18] were selected (Figure 1). Within the 12 studies, there were a total of 640 patients with CD, with 319 patients in the experimental group and 321 in the control group. One of the 12 articles [7] described the use of random sequence generation. None of the studies mentioned allocation concealment or blinding. All the patients in the study were treated with WM. The treatment group was treated with Chinese medicines combined with WM, and none of the patients were treated with Chinese medicine alone. All studies provided the baseline data. The baseline characteristics and risk of bias in qualified studies are shown in Tables 1 and 2.

### 3.2. Results of Meta-Analysis

#### 3.2.1. Total Effective Rate

Eleven studies compared the total effective rate between the two groups [7–17]. Following the tests of heterogeneity, the random-effect model could be used for all 11 studies (*P* = 0.008*, I*^2^ =58.4%). Compared with the control group, TCM combined with WM was more effective in the treatment of CD. There was a significant difference between the two groups [RR = 1.25, 95%* CI *(1.16, 1.35), z = 5.63,* P* < 0.01].

**Figure 2:**
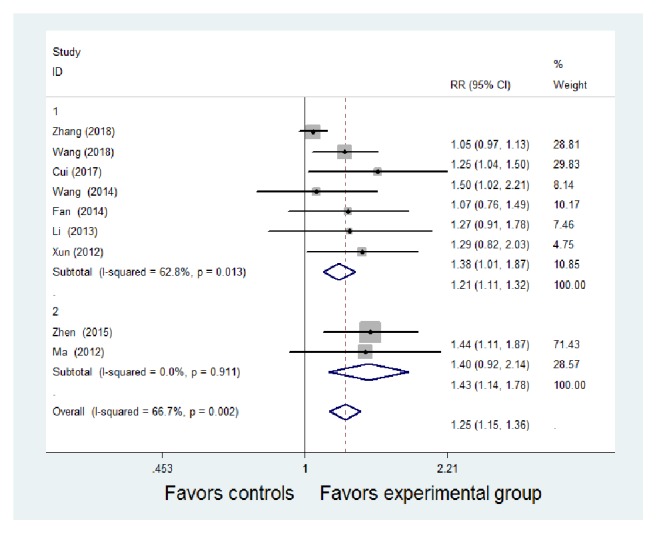
Meta-analysis of the comparison of the total effective rate between the Traditional Chinese Medicine + Western Medicine and Western Medicine groups. CI: confidence interval; RR: relative risk.

With regard to subgroup analysis according to the different curative effect evaluation standards, there were two studies [9, 11] that did not mention the specific efficacy standards and seven studies [7, 8, 10, 13–16] that adopted the standard of the Consensus on Diagnosis and Management of Inflammatory Bowel Disease by China in 2007. Meta-analysis results showed that the heterogeneity was still relatively large (*P* = 0.013 < 0.01,* I*^2^ = 62.8%). The random-effect model was used in seven studies. The results showed that TCM combined with WM had better efficacy than WM alone, and the difference was statistically significant [RR = 1.21, 95% CI (1.11, 1.32), z = 4.14,* P* < 0.01]. Two studies [12, 17] adopted the standard of the Consensus on Diagnosis and Management of Inflammatory Bowel Disease by China in 2000 (*P* = 0.911,* I*^2^ =0%); the results (*P* = 0.911,* I*^2^ = 0%) showed no obvious heterogeneity. Meta-analysis using the fixed effects model showed that the experimental group had good curative effect compared to the control group and the difference was statistically significant (RR = 1.43, 95% CI (1.14, 1.78), z = 5.19,* P* = 0.002 < 0.01] (Figure 2).

#### 3.2.2. Mucosal Healing

**Figure 3:**
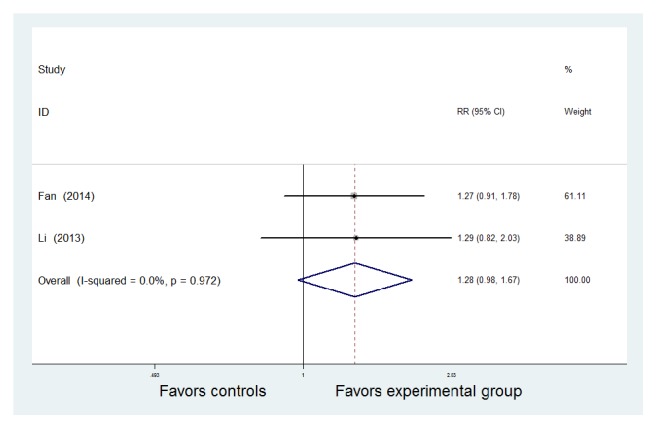
Meta-analysis of the comparison of mucosal healing between the Traditional Chinese Medicine + Western Medicine and Western Medicine groups. CI: confidence interval; RR: relative risk.

Mucosal healing was reported in two RCT studies [14, 15]. Heterogeneity testing indicated* P* = 0.972, and* I*^2^ = 0.0%, demonstrating homogeneity. Therefore, a fixed-effect model was adopted. Two studies exhibited significant mucosal healing in both groups. However, there was no significant intergroup difference between the two groups [RR = 1.28, 95% CI (0.98, 1.67), z = 1.78,* P* = 0.075 > 0.05] (Figure 3).

#### 3.2.3. Clinical Score

**Figure 4:**
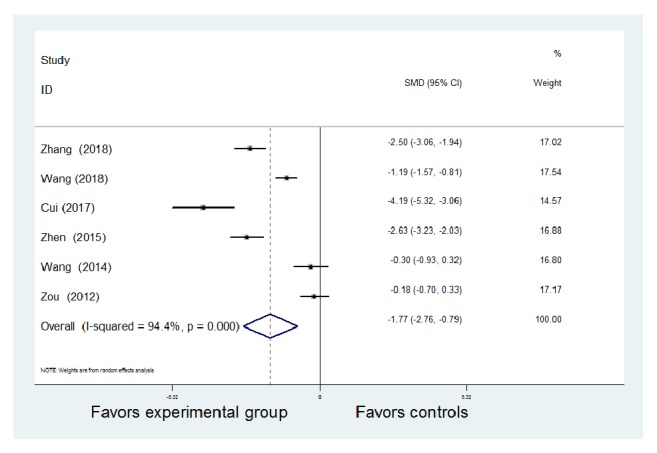
Meta-analysis of the comparison of clinical scores between the Traditional Chinese Medicine + Western Medicine and Western Medicine groups. CI: confidence interval; SMD: standardized mean difference.

The clinical score for CD was reported in six studies [7, 8, 10, 12, 13, 18]. In the six studies, the Crohn's disease activity index (CDAI) score decreased significantly in both groups. Heterogeneity testing revealed* P* < 0.01, and* I*^2^ = 94.4%, demonstrating heterogeneity. Therefore, a random-effect model was used. There was a significant difference between the TCM combined with WM and WM groups [SMD = -1.77, 95% CI (-2.76,-0.79), z = 2.91,* P *< 0.01] (Figure 4).

#### 3.2.4. CRP and ESR 

**Figure 5:**
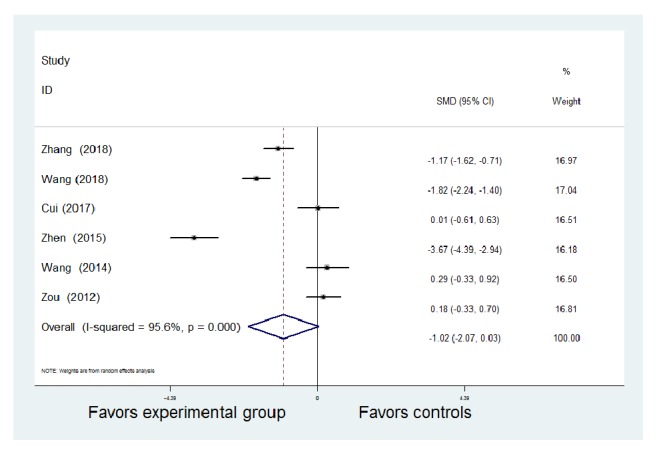
Meta-analysis of the comparison of C-reactive protein between the Traditional Chinese Medicine + Western Medicine and Western Medicine groups. CI: confidence interval; SMD: standardized mean difference.

Six studies provided data on CRP in patients with CD [7, 8, 10, 12, 13, 18]. A random-effect model was used for pooled analysis because there was statistical heterogeneity among the studies (*P* < 0.01,* I*^2^ = 95.6%). One study [8] used an immunofluoroscopy turbidimetric method, one study [7] used the ELISA method, and the other four studies did not explicitly state a measurement method. Therefore, different measurement methods may be considered as the source of heterogeneity. There were statistically significant differences in the six studies with regard to reduction of CRP levels; the experimental group showed more improvement than the control group [SMD = -1.02, 95% CI (-2.07, 0.03), z = 9.14,* P* < 0.01] (Figure 5).

**Figure 6:**
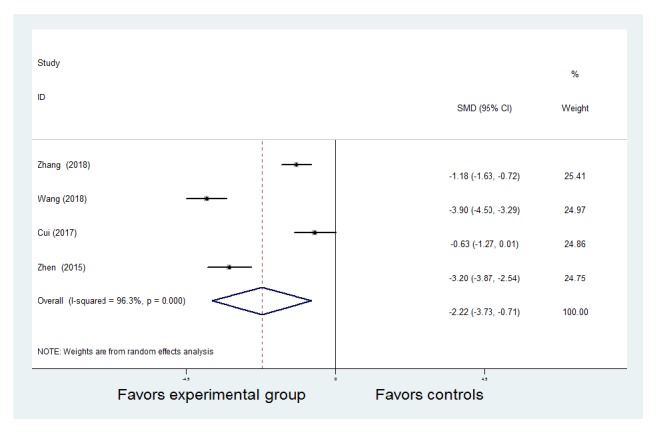
Meta-analysis of the comparison of erythrocyte sedimentation rate between the Traditional Chinese Medicine + Western Medicine and Western Medicine groups. CI: confidence interval; SMD: standardized mean difference.

ESR was mentioned in four studies [7, 8, 10, 12]. A random-effect model was used for pooled analysis because there was statistical heterogeneity among the studies (*P* <0.01* I*^2^ = 96.3%). One of the studies [8] adopted the Wechsler method, while the other three studies did not explicitly state the measurement method. Therefore, different measurement methods may be considered as the source of heterogeneity. The data showed significant reduction in ESR in both the experimental and control groups, but the experimental group had the advantage of significant reduction of ESR compared to the control group [SMD = -2.22, 95% CI (-3.73, -0.71), z = 2.89,* P* = 0.004< 0.01] (Figure 6).

#### 3.2.5. Remission Maintenance Rate

**Figure 7:**
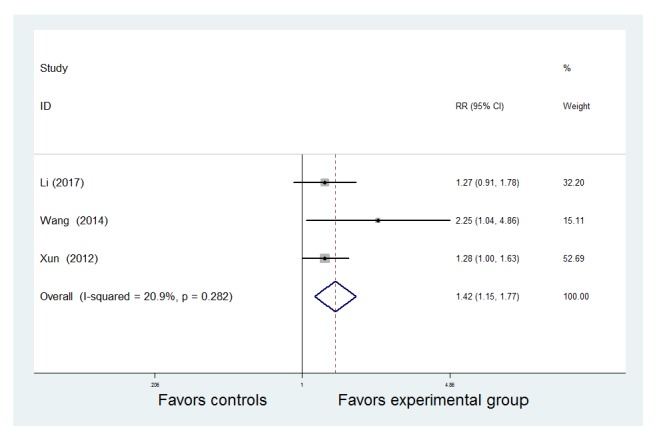
Meta-analysis of the comparison of remission maintenance rates between the Traditional Chinese Medicine + Western Medicine and Western Medicine groups. CI: confidence interval; RR: relative risk.

Only three trials provided extractable data regarding the remission maintenance rate [9, 13, 16]. As homogeneity was not important in the trial results (*P* = 0.282,* I*^2^ = 20.9%), a fixed effects model was applied. The results suggested that the number of patients who remained in clinical remission after six months was significantly higher in the TCM combined with WM group than in the WM group [RR = 1.42, 95% CI (1.15, 1.77), z = 3.20,* P* = 0.001 < 0.05] (Figure 7).

#### 3.2.6. Side Effects

**Figure 8:**
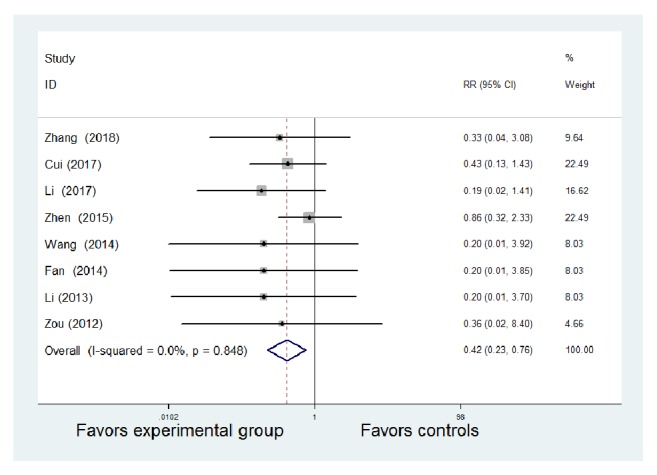
Meta-analysis of the comparison of side effects between the Traditional Chinese Medicine + Western Medicine and Western Medicine groups. CI: confidence interval; RR: relative risk.

Side effects were reported in eight studies. The eight studies provided extractable data regarding adverse events [8–10, 12–15, 18]. No obvious heterogeneity existed among the studies (*P *= 0.848,* I*^2^ = 0.0%). Therefore, we used the fixed-effect model for analysis. The incidence of side effects was significantly lower with TCM therapy combined with WM compared with WM alone [RR = 0.42, 95% CI (0.23, 0.76), z = 2.84,* P* = 0.005 < 0.01] (Figure 8).

### 3.3. Sensitivity Analysis

The results of the analysis, while eliminating each study one at a time, did not show substantial changes, indicating that the results of the meta-analysis were relatively stable.

### 3.4. Publication Bias

Egger's test and Begg's test were used to assess publication bias. The results showed no significant asymmetry of the funnel plots of the remission maintenance rate, CRP, ESR, and clinical scores (*P* > 0.05). There was significant asymmetry of the funnel plots of the total effective rate, recurrence rate, side effects, and mucosal healing (*P* < 0.05). Therefore, it can be considered that there was a certain publication bias in the included studies with regard to the total effective rate, side effects, and mucosal healing, while there was no obvious publication bias with regard to the remission maintenance rate, CRP, ESR, and clinical score.

## 4. Discussion

This systematic review and meta-analysis is the first to assimilate all the available evidence from RCTs in order to estimate the efficacy of TCM therapies for CD.

CD is one of the most refractory diseases of the digestive system. With the progress in medical technology (and increasing diagnoses made), the number of patients, and the changes in lifestyle, the incidence of CD is increasing every year. Since the pathogenesis of the disease is not completely clear, there is no good prevention strategy, and there is no specific treatment for the disease. At present, clinical treatment is aimed at controlling acute symptoms, maintaining remission, and reducing or delaying recurrence.

Traditional methods of treatment of CD focus on controlling the inflammation and regulating immune system disorders in order to effectively control disease onset and maintain remission. Although drugs such as 5-aminosalicylic acid, glucocorticoids, and immunosuppressive agents have been a big breakthrough in treatment, and are still the main drugs for the treatment of CD, the remission rate is only 70%. Even the best drugs that achieve remission can only reduce the recurrence rate by 50%, and about two-thirds of the cases eventually still need surgery. As a result, the patient's quality of life is severely affected [19].

TCM is a historically proven effective way to treat diseases under the guidance of traditional Chinese philosophy. Chinese medicine believes that the occurrence of any disease is due to the effect of pathogenic factors on the human body, causing an imbalance of Yin and Yang, and visceral and meridian dysfunction. TCM is composed of a variety of classic formulae of traditional Chinese medicines; its curative effect is achieved by adjusting the Yin and Yang of the human body and filling the weak qi and purging the win qi in the five viscera in order to balance the deficiency of Yin and Yang in the five viscera. TCM pays more attention to the unity of man and nature, the four-hour climate, and so on. In the treatment of diseases, TCM pays attention to “holistic recuperation”, while WM pays attention to “symptomatic treatment”.

The treatment of CD in Chinese medicine focuses on integrity, and has an advantage especially in the treatment of refractory CD [20]. A large number of studies have reported that integrated TCM and WM treatment can significantly reduce the clinical symptoms of CD, reduce the levels of inflammatory indicators, and promote intestinal mucosal healing. It can also reduce the recurrence rate and adverse drug reactions [21].

This meta-analysis showed that treatment with TCM-WM was more effective in terms of total effective rate, remission maintenance rate, CRP reduction, ESR reduction, clinical score reduction, and reduction of adverse events (*P *< 0.01). There was improvement in mucosal healing in both the TCM-WM and WM groups, but there was no significant difference (*P *> 0.05). Therefore, this systematic review has demonstrated a clear benefit of TCM in the treatment of CD.

However, there were some limitations in our analysis. First, we systematically evaluated 12 RCTs involving 640 patients; the sample sizes were small. Secondly, all 12 studies claimed to be RCTs, but only one described the randomization methods used in detail. None of the studies mentioned allocation concealment or blinding. Two studies reported the number of patients who were lost to follow-up and dropouts, and used intention-to-treat analysis. Additionally, the quality of evidence was generally rated as moderate by the GRADE criteria and the risk of bias was moderate. Therefore, the poor methodological quality of the studies may have affected the results. Finally, although we thoroughly screened the English database, there were no English studies. Publication bias may also exist in the study. Studies on efficiency, scoring, ESR, and CRP were relatively heterogeneous. Subgroup analysis was used for clinical efficacy, and the results showed that the evaluation criteria for clinical efficacy were different, which may be the source of heterogeneity. Regarding ESR, CRP, and clinical scoring, since some studies did not provide sufficient clinical data, we could not conduct in-depth analysis on the sources of heterogeneity. Heterogeneity may be due to differences in reagents and methods used for measurement, measurement units, clinical baseline characteristics included in the study, and intervention programs.

## 5. Conclusions

The current data indicate that TCM combined with WM is more effective than WM alone in terms of inducing remission in CD, especially with regard to effective rate, remission maintenance rate, CRP reduction, clinical score reduction, and reduction of adverse events. Our results suggest that adjunctive treatment with TCM may have better therapeutic effects in CD. Because of the poor quality of the included studies, the conclusion should be interpreted with care. Accordingly, more randomized, double-blind, sham-controlled studies are needed to assess the clinical value of TCM in the treatment of CD patients.

## Figures and Tables

**Table 1:** Characteristics and methodological quality of included studies.

Study ID	Sample size (E/C)	Sex		Age (E/C)	Therapy		Duration (weeks)	Outcome
		M(E/C)	F(E/C)		Experimental	Control		
Zhang 2018	44/44	24/26	20/18	38.6±7.2 (23~58)/36.9±8.3(25~54)	TCM+Prednisone	Prednisone	8	Efficacy, ESR, CRP,CDAI, Side effects
Wang 2018	62/62	36/34	26/28	37.10±5.68(21~65)/36.61±5.29(19~65)	TCM+Thalidomide	Thalidomide	12	Efficacy, ESR, CRP,CDAI
Cui 2017	20/20	11/10	9/10	42.25±15.37/35.25±14.46	TCM+Mesalamine	Mesalamine	12	Efficacy, ESR, CRP,CDAI, Side effects
Li 2017	15/15	10/9	5/16	28.4 ±3.8(22~46)/27.8±3.4 (20~42)	TCM+ Sulfasalazine	Sulfasalazine	24	Efficacy, Side effects, Recurrence
Deng 2016	30/30	- - - -	- - - -	_	TCM+ Mesalamine	Mesalamine	12	Efficacy
Zhen 2015	40/40	23/21	17/19	37.8 ±6.5(21~63)/36.8±5.7(19~65)	TCM+ Methotrexate	Methotrexate	8	Efficacy, ESR, CRP,CDAI, Side effects
Wang 2014	20/20	12/11	8/9	35.70±13.07(18~60)/37.85±12.42(18~65)	TCM+ Mesalamine	Mesalamine	24	Efficacy, ESR, CRP, Recurrence
Fan 2014	15/15	- - - -	- - - -	(18~65)	TCM+Prednisone	Prednisone	24	Efficacy, ESR, CRP, CDAI, Mucosal healing
Li 2013	10/10	7/6	3/4	35 ±13(18~65)/38±10(18~65)	TCM+Prednisone	Prednisone	24	Mucosal healing
Xun 2012	24/24	- - - -	- - - -	28.12±2.57(18~45)	TCM+Sulfasalazine	Sulfasalazine	24	Efficacy, Recurrence
Ma 2012	16/16	- - - -	- - - -	28.12±2.57(18~45)	TCM+Prednisone	Prednisone	10	Efficacy
Zou 2012	28/30	15/16	13/14	35.89±11.49(15~70)/34.29±17.91(15~70)	TCM+ Sulfasalazine	Sulfasalazine	4	ESR, CDAI

Notes: CDAI: Crohn's disease activity index; CRP: C-reactive protein; ESR: erythrocyte sedimentation rate; E: experimental group; C: control group; TCM: Traditional Chinese Medicine; WM: Western Medicine.

**Table 2:** Quality assessment of included randomized controlled trials.

Study ID	Assessment of methodological quality of the studies						
	1	2	3	4	5	6	Grade of risk bias
Zhang 2018	U	U	N	Y	Y	U	B
Wang 2018	U	U	N	Y	Y	U	B
Cui 2017	U	U	N	Y	Y	U	B
Li 2017	U	U	N	Y	Y	U	B
Deng 2016	U	U	N	Y	Y	U	B
Zhen 2015	U	U	N	Y	Y	U	A
Wang 2014	Y	U	N	Y	Y	U	B
Fan 2014	U	U	N	Y	Y	U	B
Li 2013	U	U	N	Y	Y	U	B
Xun 2012	U	U	N	Y	Y	U	B
Ma 2012	U	U	N	Y	Y	U	B
Zou 2012	U	U	N	Y	Y	U	A

Notes: 1: randomization; 2: allocation concealment; 3: blinding; 4: complete outcome data; 5: selective reporting of outcomes; 6: other bias; Y: yes; N: no; U: unclear.
